# QTREDS: a Ruby on Rails-based platform for omics laboratories

**DOI:** 10.1186/1471-2105-15-S1-S13

**Published:** 2014-01-10

**Authors:** Piergiorgio Palla, Gianfranco Frau, Laura Vargiu, Patricia Rodriguez-Tomé

**Affiliations:** 1 Center for Advanced Studies, Research and Development in Sardinia (CRS4), Loc. Piscina Manna, Pula 09010, Italy; 2 Department of Electrical and Electronic Engineering (DIEE), University of Cagliari, Cagliari 09123, Italy; 3 Department of Life and Environmental Sciences, University of Cagliari, Cagliari 09042, Italy

## Abstract

**Background:**

In recent years, the experimental aspects of the laboratory activities have been growing in complexity in terms of amount and diversity of data produced, equipment used, of computer-based workflows needed to process and analyze the raw data generated. To enhance the level of quality control over the laboratory activities and efficiently handle the large amounts of data produced, a Laboratory Management Information System (LIMS) is highly-recommended. A LIMS is a complex software platform that helps researchers to have a complete knowledge of the laboratory activities at each step encouraging them to adopt good laboratory practices.

**Results:**

We have designed and implemented Quality and TRacEability Data System - QTREDS, a software platform born to address the specific needs of the CRS4 Sequencing and Genotyping Platform (CSGP). The system written in the Ruby programming language and developed using the Rails framework is based on four main functional blocks: a sample handler, a workflow generator, an inventory management system and a user management system. The wizard-based sample handler allows to manage one or multiple samples at a time, tracking the path of each sample and providing a full chain of custody. The workflow generator encapsulates a user-friendly JavaScript-based visual tool that allows users to design customized workflows even for those without a technical background. With the inventory management system, reagents, laboratory glassware and consumables can be easily added through their barcodes and minimum stock levels can be controlled to avoid shortages of essential laboratory supplies. QTREDS provides a system for privileges management and authorizations to create different user roles, each with a well-defined access profile.

**Conclusions:**

Tracking and monitoring all the phases of the laboratory activities can help to identify and troubleshoot problems more quickly, reducing the risk of process failures and their related costs. QTREDS was designed to address the specific needs of the CSGP laboratory, where it has been successfully used for over a year, but thanks to its flexibility it can be easily adapted to other "omics" laboratories. The software is freely available for academic users from http://qtreds.crs4.it.

## Background

The rapid development of high throughput sequencing and microarray technologies in the last years and the simultaneous introduction of the so-called next-generation sequencing instruments have produced two main consequences: a large amount of data generated, new and complex laboratory procedures [1-3].

A Laboratory Management Information System (LIMS) is designed considering the need to carry out the research in an efficient and transparent manner allowing the implementation of different quality control strategies and improving the accessibility of the instruments. The improvement of the laboratory activities involves three primary factors: technology, information and people. In order to develop an effective LIMS all the three resources must be recognized and a thorough study of the laboratory processes must be taken into consideration.

Till the late 1970s all the activities concerning the management of laboratory samples, associated analysis and reporting were time-consuming and error prone due to manual processes [4]. This gave some organizations impetus to optimize data collection and laboratory procedures. Initially some custom in-house solutions have been developed, while some analytical instrument manufacturers, at the same time began to develop some commercial systems to run on their instruments.

The term LIMS entered the commercial world in the early 1980s to describe systems used in the pharmaceutical and related industries as Quality Assurance and Quality Control tools [4,5].

In 1982 the first generation of LIMS was introduced in the form of single centralized minicomputer provided with automated reporting tools. Second generation LIMS became available in 1988 and used third-party commercial relational databases to provide application-specific solutions. Most of them relied on minicomputers [6]. Third generation LIMS began in 1991, as personal computers became more powerful and prominent. They combined the personal computer's easy to use interface and standardized desktop tools with the computational power and reliability of minicomputer servers in a client/server configuration. By 1995 fourth generation LIMS came into the picture decentralizing the client/server architecture further, optimizing resource sharing and network throughput by enabling process to be performed anywhere on the network [4].

From 1996 to 2002 additional features and functionalities were included in LIMS, from wireless networking capabilities and geo-referencing of samples, to the adoption of XML standards [4].

In the latest generation LIMS the adoption of web oriented software technologies assumes a key role [2] together with a rising interest in the Software as a Service (SaaS) distribution model through which the customers can save the expense of license fees and the costs of hardware and maintenance.

In this paper, we propose QTREDS, a software platform initially born to address the specific needs of the CRS4 sequencing laboratory. The main purpose of our in-house solution was to set up a system that provides the researchers with a complete knowledge of the laboratory processes at each step, managing and verifying the:

• workflow creation;

• samples traceability;

• diverse experimental protocol definitions;

• inventory of reagents;

• users' roles and privileges.

Why develop an in-house LIMS rather than buy a commercial one? A great number of proprietary LIMS have been developed. STARLIMS [7], Exemplar LIMS [8], LABVANTAGE SAPPHIRE [9] just to name a few, allow customers to benefit from vendors long-established experience and valuable resources.

On the other hand, most often these commercial solutions are large, complex and feature-rich products designed to be sold to large laboratories. Their license fees are usually prohibitive and each extra feature or module they provide might come at additional costs [10]. Furthermore the laboratories have to buy also the servers, peripherals, storage devices and other software licenses (such as databases, load balancers, etc...). Most small or medium-sized laboratories cannot afford this expense [11].

Many commercial LIMS vendors are now offering rented, hosted and SaaS-based LIMS solutions. The rental approach is almost identical to the purchased one, except that the laboratory rents the software rather than purchasing the license. All the other purchases (hardware and additional software) remain the same, as do other costs [11]. The major difference is a staged payment for the software.

Some LIMS vendors provide hosted thin-client solutions. A thin-client LIMS is an architecture which offers full application functionality that can be accessed through a simple web browser. Rather than requiring the customer to purchase hardware and software, the customer simply uses the software running at the vendor's site. However, hosted software providers often do not rewrite their products to take advantage of new Internet-based technologies, but simply put a different front-end onto dated systems.

Another approach is the cloud-based model. While it bears some resemblance to the hosted model, the cloud-based SaaS model is usually built from the ground up using a service-oriented architecture. They are designed for multi-tenancy, where multiple customers share the same instance of the application running on the same operating system, on the same hardware, with the same data-storage mechanism.

These software applications are designed to virtually partition their data and configurations, so that customers do not see each other's data and work within their customized virtual application instances. According to this model, data are stored on the servers of the service provider and this fact can raise a number of issues if data confidentiality is critical, as it often happens in the biomedical field [11,12].

Many open-source LIMS are now available, but some of them had not been published when we started the development of QTREDS in early 2011 [12-14].

Before starting the development phase, we tried some of the solutions available at the time: we tested Open-LIMS [15] by installing it on our server but it was very unstable, in fact it was not recommended by the developers to use it in any productive environment; we also tried Bika Lims [16] which is one of the leading open source LIMS, with a wide range of applications from agriculture to environmental monitoring. It offers many functionalities for free, but optional modules at a cost. It is based on Python and the Plone content management system. We programmed web services with Python Zope and Plone, and our experience is that it is not a trivial software stack. Furthermore Plone performs better on a dedicated server and that could represent an hidden cost. Other systems we have looked at, but not considered because we felt they did not correspond well to our needs are: LabKey Server [17] a very much oriented to data analysis tool. In our case, the experiments are done "as a service", and the results are given to the researchers. The analysis are not done in the laboratory.

SLIMS [18] a Sample-based Laboratory Information Management System with a web-based interface to create, edit and view sample information. SLIMS is designed to store and manage biological data in fact it features a micro-plate annotation tool and supports SDS-PAGE gels. It can also generate and export reports, but it does not provide any inventory management system and its web interface does not include the latest web technologies.

GNomEX [1] a very complete platform that includes a next generation sequencing/microarray LIMS, an analysis project center, and an application for annotating and programmatically distributing genomic data. It is much more complex than QTREDS and it was designed for large research centers and clinics. Because of that it does not meet the needs of a relatively small entity like the CSGP laboratory. We tested also other solutions, but some of them were in a very early development stage or they were buggy and crashing and not stable enough to run in a production environment [14,19].

The most important factor for the development of an in-house solution, even more than the license fees or the confidentiality issues, was the fact that the application had to be developed to meet the specific needs of the researchers of the CSGP laboratory. When we started to develop QTREDS, the main project in our laboratory was related to the DNA sequencing of 2100 individuals from Sardinia [20]. At the same time other projects concerning RNA and exome sequencing of a large part of the same set were in their early stages.

While for the DNA sequencing the techniques and procedures in use were well defined and standardized, in the case of RNA and exome sequencing, the methodologies and the protocols had not been decided yet, so we began to develop QTREDS not only to collect the data, trace and manage each lab activity, but also to help researchers choose the best protocol to implement for their experiments.

We designed a system flexible and responsive enough to keep up with the speed at which the laboratory evolves.

## Implementation

### Development methodologies

QTREDS has been developed adopting an Agile software development approach. Indeed, we have worked closely and continuously with researchers, operators, managers and other stakeholders involved in the project. In particular, we followed a Behavior-Driven Development (BDD) strategy, asking questions focused on the behavior of the platform before and during the development stages, to avoid or at least reduce misunderstandings between stakeholders.

Requirements were written down in the form of user stories, which described the expected use of each part of the application. User stories, a lightweight approach to use case analysis, have been compiled and continuously refined, in nontechnical language allowing all stakeholders to be involved in the process of creation and prioritization of the requirements.

Starting from a general description of the needs of the CSGP laboratory and the main functional requirements that the system was expected to have, we created a working but incomplete prototype, refining it constantly through a continuous interaction with the researchers and the personnel of the laboratory until the achievement of the desired results.

### Software architecture and design patterns

QTREDS is a web application with a client-server architecture developed in the Ruby programming language, using the framework Rails [21].

**Figure 1:**
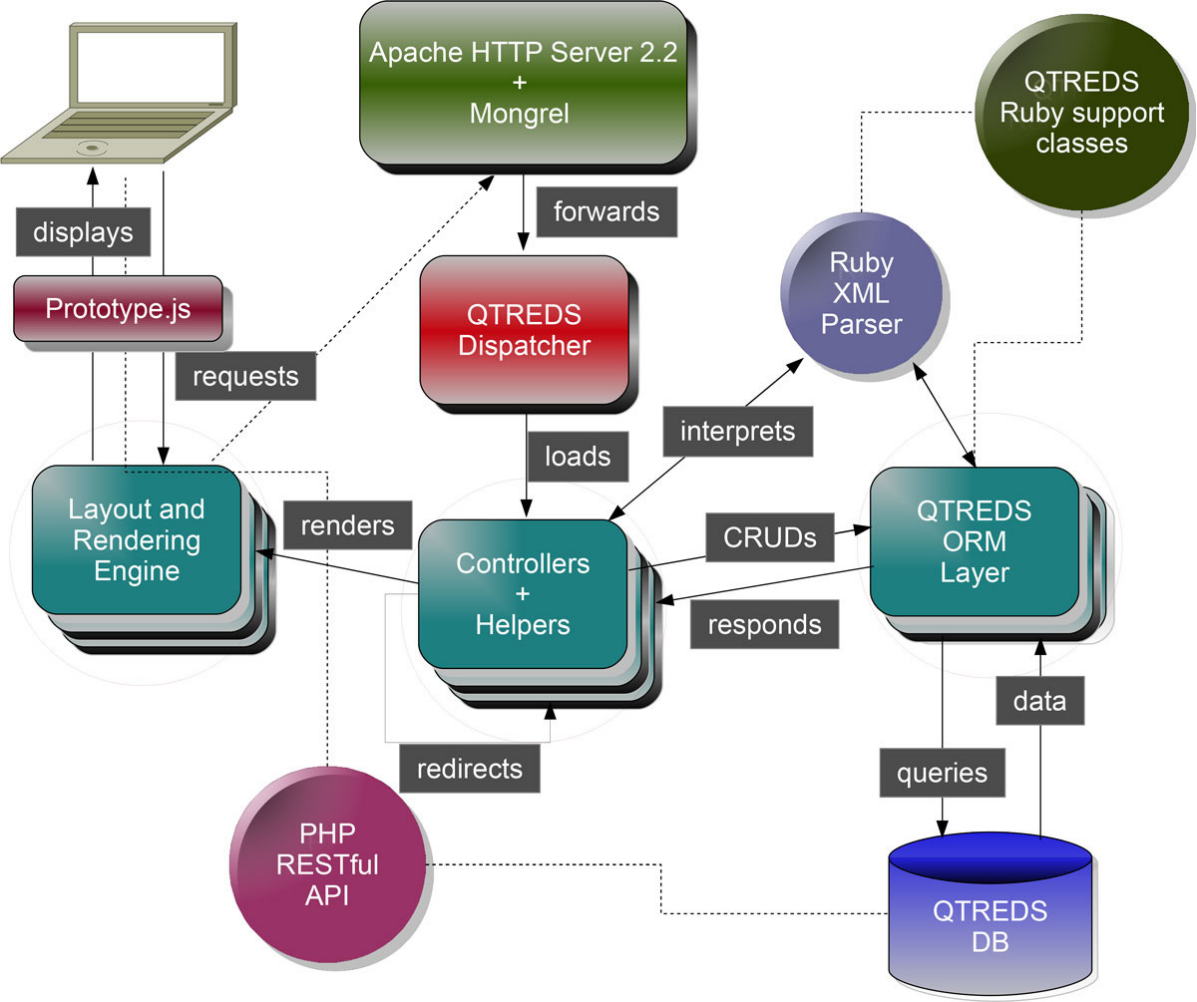
**QTREDS architectural overview**. QTREDS has been developed according to the MVC software architecture pattern. The Protocol Parser and the internal class libraries have a key role for the generation of the experimental workflows.

The application, according to the architectural pattern known as Model-View-Controller (MVC), has been organized dividing the code into three kinds of components (Figure 1). Models implement business logic and are concerned with the manipulation of the data: how to store it, to change it or move it. Typically for each type of entity managed by the application, we have created a corresponding model that encapsulates it. Views serve as the interface between application users and model data. They contain information about the models with which users can interact and manage how to display it. Controllers have the role of intermediaries between views and models in both directions: when a user interacts with a view, a precise controller action corresponding to that activity is invoked and it saves or updates data from the user to the model. On the other hand, the controller makes the model data available to the view so that it can be displayed to the user.

One important job of the Model is to persist data which requires that some correspondence must be established between the operations on a model object in memory and how it is manipulated in the storage tier.

Models implement the Active Record architectural pattern, providing an Object Relational Mapping (ORM) layer which supports a wide variety of Relational Database Management System (RDBMS). For the QTREDS persistence tier we have chosen the MySQL RDBMS [22]. Each instance of a model class corresponds to a single row in a specific table of the MySQL database. The model object has built-in behaviors that allow to directly operate on the tables of the storage layer of the application.

The implementation of QTREDS also relies on the use of different open-source programming libraries. The web user interface has been developed combining the Ruby's built-in erb templating system with the Prototype JavaScript Framework [23] that enabled us to deal with the Asynchronous JavaScript And XML (AJAX) [24] technology in a very easy and efficient way.

Furthermore the use of the script.aculo.us [25] set of Javascript libraries provides us with a visual effects engine, that we used to enhance the interactive user experience with the application.

## Results

### Functional overview

All the activities and operations allowed by the QTREDS platform can be assigned to four different functional blocks: 1) workflow management system, 2) sample handler, 3) inventory management system, 4) authorization system.

### Workflow management system

**Figure 2:**
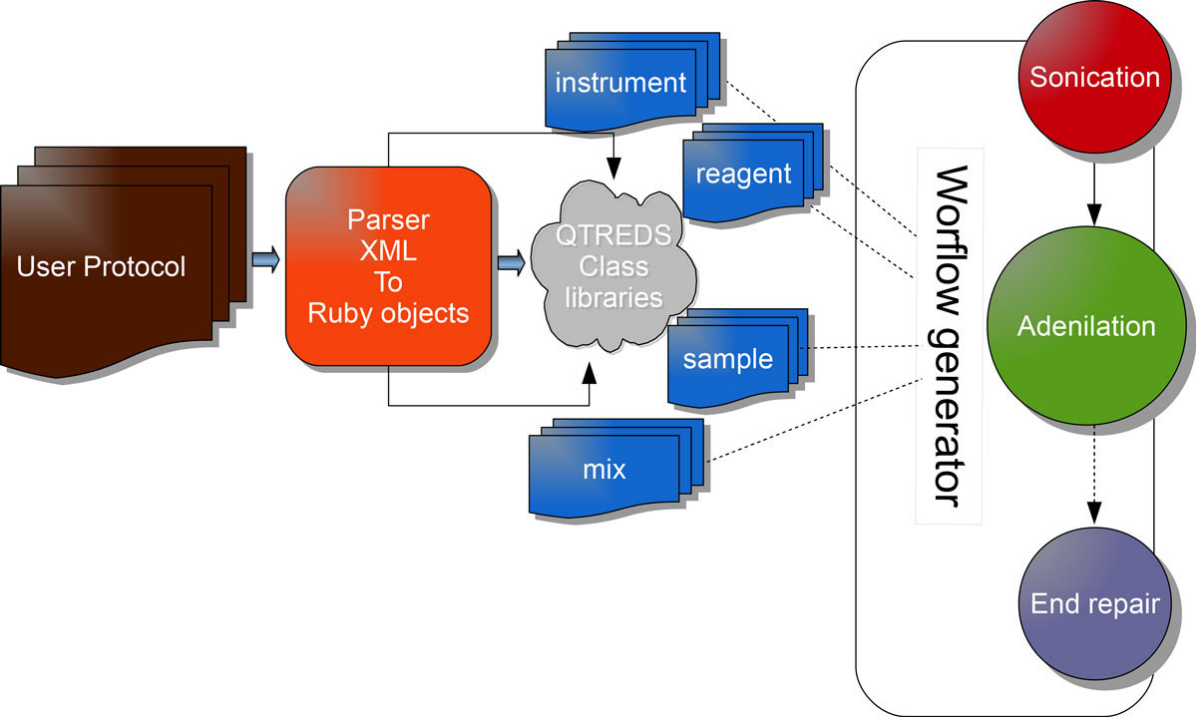
**Workflow management system - the protocol parser**. The parser checks and interprets the experimental protocols written in the XML format and with the help of the internal class libraries provides the controllers with the information needed to generate the experimental workflow.

The workflow management system is a key component of our application. Figure 2 illustrates the main concepts related to this functional block: it has the responsibility for defining and verifying a protocol and to convert it into the sequence of steps and tasks that represent a particular procedure or experiment.

A protocol, in our system, is a formal representation of an experimental procedure, expressed in the XML language (see Additional file 1 for a concrete example) that has to be compiled according to a strict set of rules that we defined and collected in an XML Schema Definition (XSD) document (see Additional file 2 for a complete description).

This task can be accomplished manually by an authorized member of the laboratory with basic informatics knowledge. But writing down a protocol manually can be a very long, boring and error-prone task that requires the observation of precise syntactic and semantic rules. To reduce the probability of error and to allow users with no technical background to create an experimental protocol, we have developed a user-friendly visual tool, which we describe later in this article.

**Figure 3:**
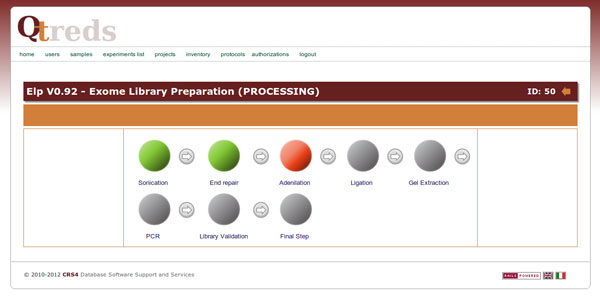
**Experiment workflow**. QTREDS represents the experiment workflow as a sort of state diagram that guides the user step by step, enabling him to perform and monitor each phase of the experiment.

**Figure 4:**
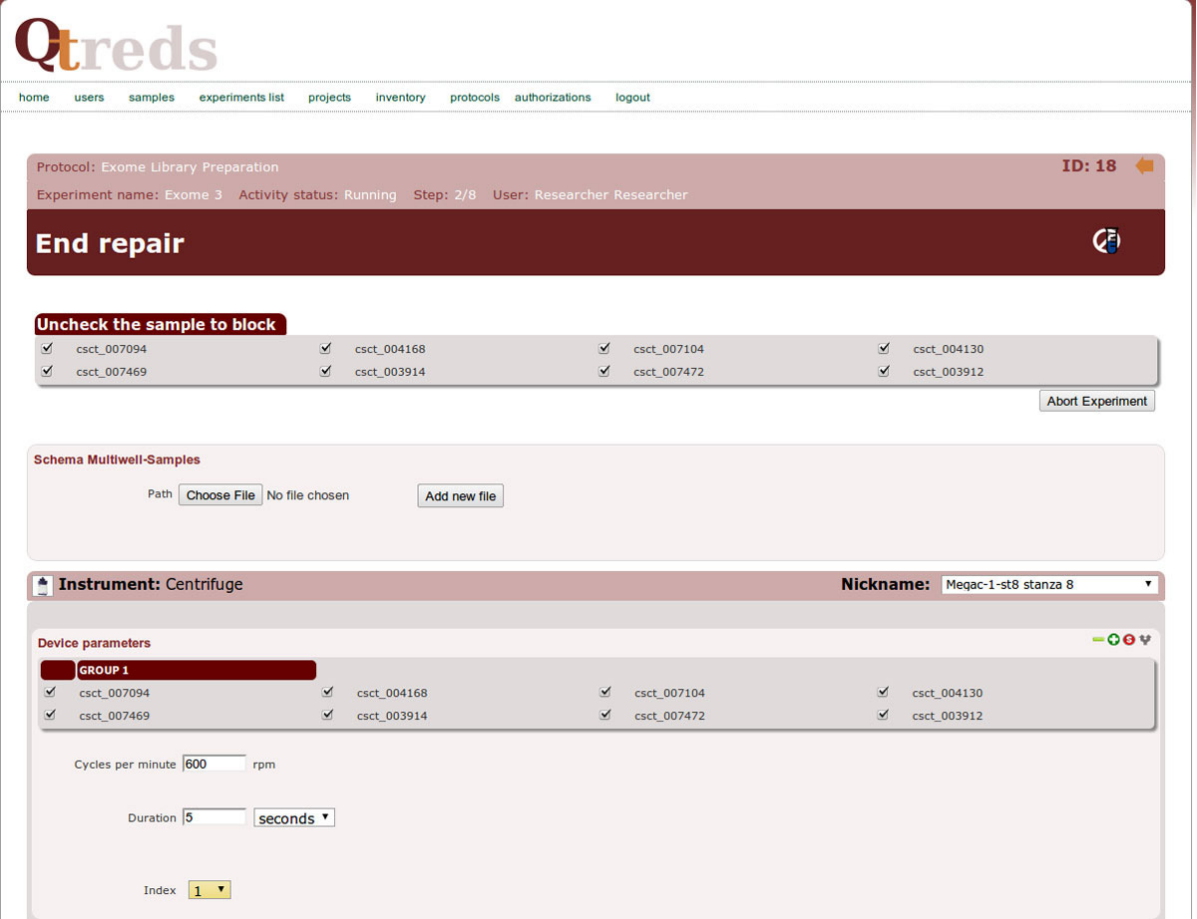
**A single step of the workflow**. When a user clicks on one of the circles of the workflow diagram he will get a web page with forms to enter data and information related to the particular step represented by that circle.

The XML protocol is interpreted and checked by the protocol parser module that processes the document, extracting and sending information to some support classes. Coordinating the activities of these classes and of the experiment-related controllers and views, it provides the system with all the information needed to graphically represent the experiment workflow as a sort of "state diagram" that guides the user step by step, enabling him to manage and monitor the progression of his experiment. Figure 3 illustrates the steps of an exome library preparation workflow of a running experiment. Exome library preparation is one of the procedures performed within the exome sequencing technology. The workflow is graphically represented as a sequence of different color balls. Each labeled ball describes a single step of the laboratory procedure (sonication, end repair, adenilation, etc...) and its color defines its state: a green ball represents a completed activity, an orange ball an activity ready to be executed or in progress and not completely carried out; a red ball indicates that the corresponding activity has been terminated abnormally for some reason, and that the workflow cannot be carried out. Grey balls represent steps of the workflow not yet available that require the completion of previous activities to be performed (Figure 3). When a user clicks on the ball of the step he wants to begin - order as we said, is mandatory at the moment - he will get a web page with forms to enter data and information related to that particular step (Figure 4). If default values have been set in the protocol/workflow definition then these will be already filled in the form. The user will then only have to fill what is different from the default, and then start the process. The complexity and level of detail of each of these web pages depends on how the users have defined that step in the protocol: it can be general or describe precisely every single phase of the process. It is up to the "workflow supervisor", i.e. the user authorized to create workflows, to decide the level of granularity of the information and the community standards to be used (e.g., MIBBI [26], ISA-Tab [27]).

Workflows can be created directly in XML or using the Visual Tool. Plugins can be implemented for the Visual Tool to check for the required information of the chosen standard. Plugins can also be written to export the data to various formats for inclusion in submissions to public databases. None of the laboratories we are collaborating with, is equipped with robots that can transfer samples and reagents between machines; because of that human intervention is always required between different steps of the workflow. So far the workflows that have been implemented reflect this and do not automatically activate the next step.

### Visual tool

Whenever an authorized user creates a new protocol he has to upload the related XML protocol description file to the system. At this point, the system checks the file for syntactical correctness and semantic coherence and it stops when the document does not follow the rules defined in the XSD document.

**Figure 5:**
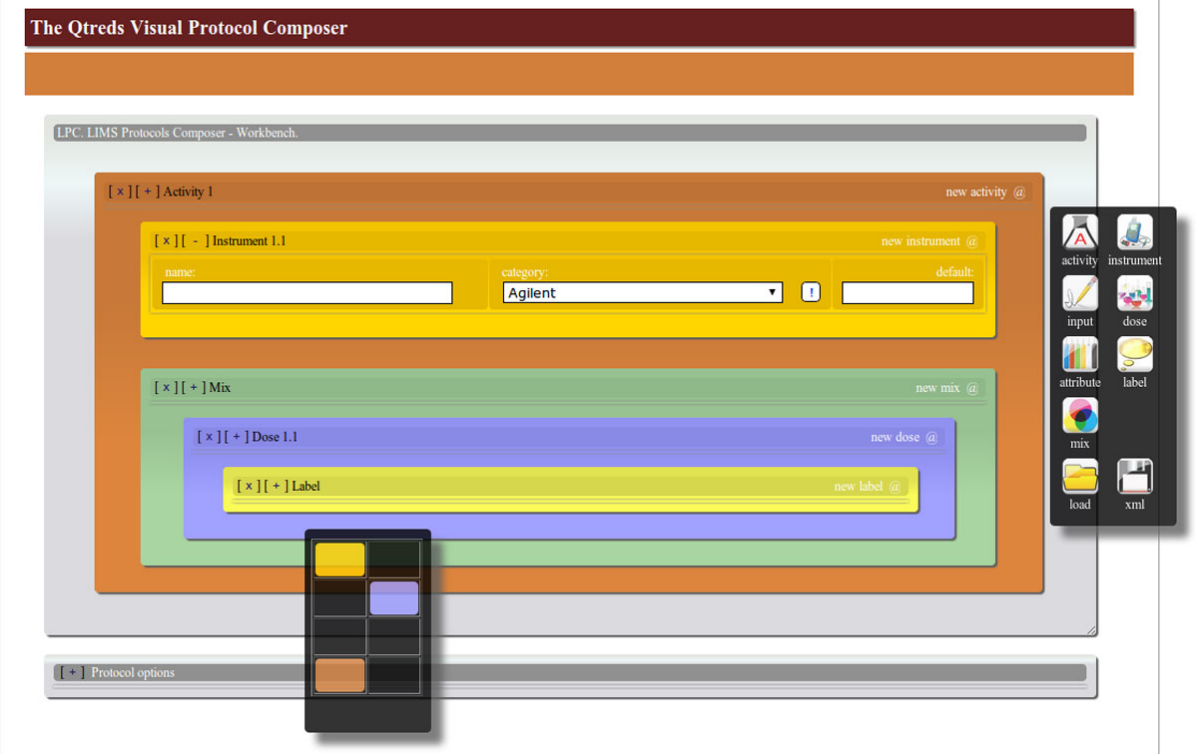
**Overview of the graphical user interface to design experimental protocols**. The visual tool allows the user to drag graphical objects from the right-most floating palette and drop them on the workbench. Combining those objects the user can create experimental protocols and export them to XML files.

As already mentioned, the process of defining and writing down an experimental protocol can be very complex and annoying. In order to simplify this task, we developed a special tool for creating protocols: it allows the user to drag-and-drop graphical objects to create experimental protocols in the XML format (Figure 5). Each visual object has the aspect of a box and can be filled up with other objects, according to the rules defined in the XSD document. This reproduces the hierarchical structure of the XML protocols written down manually.

The interface is made up of two main components: a workbench in which the user can combine all graphical elements, and a floating palette in which he can find different elements needed to define an experimental protocol: an *activity *object, that represents a single step of an experiment, an *instrument *object, which can identify any device or machine present in the laboratory, a *dose *object to describe a particular reagent to use and so on. The user can combine all these elements, organize them in the appropriate hierarchical order and set all the parameters that are needed. The result of this graphical representation can be easily exported in the XML format and used by the workflow management module of the system. Through the visual tool the user can also import an existent XML protocol, convert it to a graphical representation and manipulate it with the editing tools provided.

The tool has been implemented in pure HTML5 and JavaScript. HTML5 defines an event-based mechanism and additional markup for natively supporting drag and drop operations. This allowed us to develop a faster and more responsive tool, without the support of any other JavaScript library or framework.

### Sample handler

QTREDS enables the users to enter either one single sample or multiple samples at a time using an Excel spreadsheet-based wizard. In the first case the user should fill in a web form providing some mandatory information, for instance a unique sample identifier (*sample id*).

In the second case, the user loads a group of samples through an Excel file: the wizard allows the mapping of each column of the spreadsheet to one of the attributes used by the system to describe a sample. After a sample is entered into the system, a new record is saved to the database with its defined set of attributes.

If the number of columns of the spreadsheet mapped exceeds the number the samples' attributes or if the user needs to associate a sample with some extra parameters, the system will store them in a different table. To characterize each sample, we have defined two attributes, the *original id *that corresponds to the identifier with which a sample is submitted to the laboratory and the *lab id *that is an internal parameter used by the system for the sample tracking process. Samples may be inputs of an experiment in which they are processed to generate new samples. The output samples created, keep their relationship with the inputs, holding the same *original id *value, while they change their *lab id *in relation to the particular experiment in which they were involved.

QTREDS checks for the uniqueness of the combination of the two attributes, refusing samples with the same *original id *and *lab id*.

**Figure 6:**
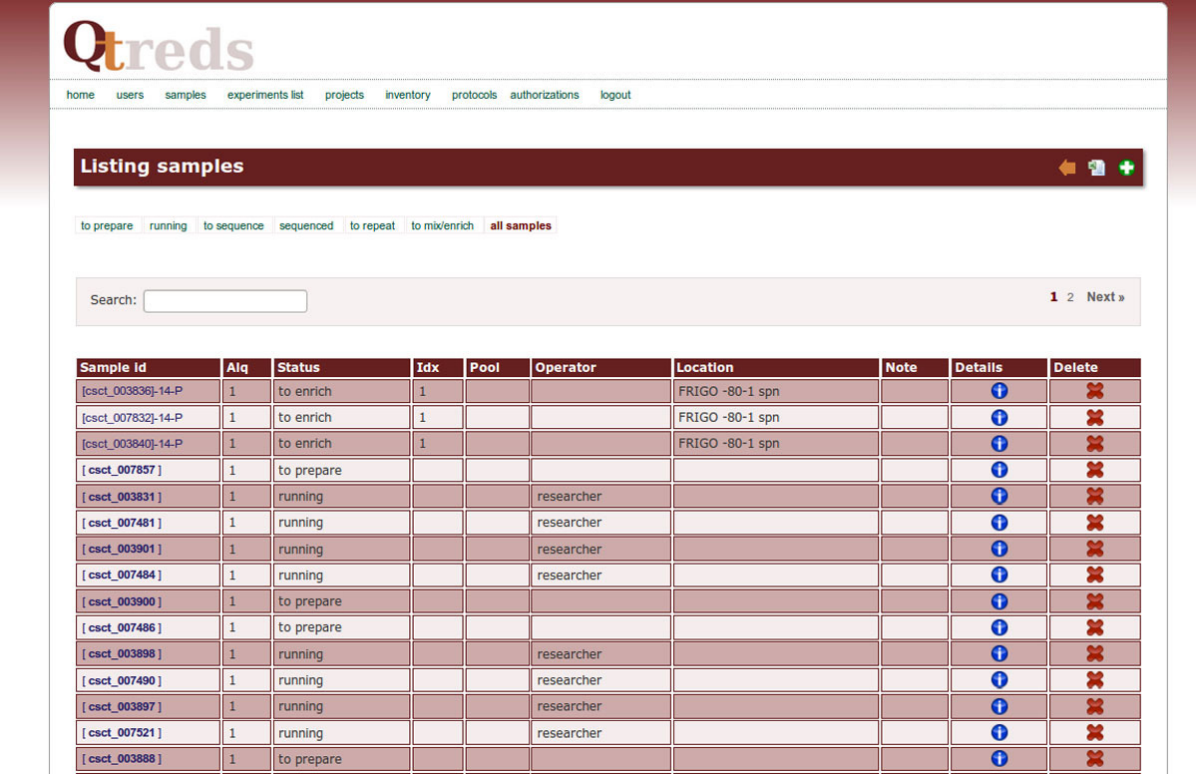
**Sample management**. Each experimental protocol modifies the status of the samples associated. Exploiting the value of the state attribute of the samples QTREDS identifies which processing activity can be done and the class of experiment each sample can be associated with.

Depending on the experimental procedure carried out, the system internally associates to each sample an attribute called *state*, which describes the current status of the sample (for instance, a DNA sample could be processed to construct a DNA library; in this case the value of the attribute *state *will change from "untreated" to "library"). The value of this attribute is exploited by the system to identify which processing activity can be done and the class of experiment each sample can be associated with (Figure 6).

### Inventory management system

The Inventory Management module allows the tracking of all the reagents and items used by the researchers for their experiments. It includes four different components:

• *catalog*: all items (consumables, reagents, tubes, etc...) involved in some laboratory process, are represented in QTREDS as abstract entities that we defined as categories. A category is not a physical item that can be found inside the laboratory, but it is an abstract description of a set of objects that share some features. The catalog collects all these categories, allowing the basic CRUD operations on them;

• *stock*: the smallest physical instance or unit of a particular category is referred to as stock. A stock indicates an item physically present in the laboratory and it specifies its quantity. To prevent the danger that a running experiment may be interrupted due to shortage of reagents or other consumables, the system provides a mechanism of "real time" assessment of stock levels, warning the researchers if some item goes below a defined threshold;

• *personal stock*: before starting an experiment, QTREDS lists all reagents and consumables needed to conduct it. The personal stock is a sort of "shopping cart" in which each researcher must insert all the items required to perform his experiment. Each experiment is represented as a sequence of consecutive steps called *activities*. The system does not allow the user to begin his experiment if his personal stock does not contain at least the reagents needed to perform the first activity.

• *topology*: starting from a simple YAMAL file, QTREDS builds a hierarchical map of the laboratory modeled as a rooted tree. The root of the tree is the whole laboratory, the subsequent nodes are the different rooms, then the freezers, going down to the granular level of the shelves, racks, etc. This representation is used by the system to track sample location.

### Authorization system

QTREDS is a web-based multi-user application. Many users can access the system simultaneously, define their own projects, experiments and manage the inventory. Within this context, it is very important the definition of user privileges and roles. The authorization module defines different user roles, each with a different access profile; each role includes a set of features and privileges to which the assigned user have access. So far, we have implemented six main roles: administrator, supervisor, simple user, inventory manager, analyzer and viewer. Depending on the role assigned, each user is allowed to perform different levels of operations and access different kinds of information. For example a simple user can see only data related to his experiments or to the projects in which he is involved, while the administrator has a complete view of all the activities and data processing operations in the laboratory. A user can have different roles in different projects. The core of the authorization module includes a set of database tables in which is stored all of the information about user roles and privileges, and a centralized authorization function. This function provides access rights and privileges to each user according to:

• user identity (*user_id*);

• specific action to be performed (*auth_id*);

• some additional parameters.

The response this function returns can be a boolean value, which tells if the user is allowed or not to perform that action or a SQL query that is used by the system to extract all the information a user can access to, according to his role.

Each user's request to gain access to a specific resource, involves a call to the centralized authorization function, passing along some arguments (for example, the *user_id *and the *auth_id*) to it. To retrieve these parameters, the system has to perform some queries on those database tables that are related to the authorization mechanism. In order to reduce duplicate queries and repeated function calls, we have implemented a caching strategy that allowed us to improve the performances of the system in terms of responsiveness and reactivity.

## Conclusions

QTREDS has been developed, starting from the local needs of the CSGP [28], where it has been used since late 2011 to make almost one hundred DNA library preparation and sequencing experiments, processing thousands of samples. We received two different kinds of reaction from the users of the QTREDS system: the ones working in team fully adopted the tool for their daily activities, providing us continuously feedback for the development of new features; on the other side some of those working on individual assignments had more difficulties to accept it.

A positive point for the users of QTREDS has been the fact that it has an iPad optimized user interface: all users of our laboratory are equipped with tablets and can enter data into our system as they would with a paper notebook while moving around for the experiments. Another point of satisfaction has been the implementation of the "personal stock" tool in the inventory management system. It warns users about all consumables and items needed, helping them in the smooth run of the experiments by preventing an abrupt stop due, for example, to lack of a given reagent. When a first demo version of QTREDS was released, some users complained the absence of simple computational tools to convert measurement units or to calculate some common physical quantities like mass, concentration and so on.

The requests have been addressed and satisfied in a later stable release introducing new elements and attributes in the XSD file and enriching the XML protocols with new functionalities.

As a whole, most of the users appreciated the way QTREDS improved the management of information especially when there was a huge increase of the number of samples being treated.

A new version of QTREDS is currently being tested and it is going to be released. The upcoming version is provided with an efficient Application Programming Interface (API) in order to allow a smart and automated access to information. The API has been implemented according to the REpresentational State Transfer (REST) architecture [29]. Using this API any authorized user or system can retrieve resources and information via a standard Hypertext Transfer Protocol request, appending the appropriate query parameters to the URL.

The RESTful web service, based on a dedicated web server, handles requests from clients, processes and then returns the appropriate response as an XML document. The API can also be used to insert data into the QTREDS database tables, creating this way, a bidirectional communication channel between our system and any other external application or tool. The new release will also provide a complete reporting system to visualize and export data in different file formats.

Thanks to its flexibility our system can be easily adapted to address the issues and the needs of other kinds of laboratories; therefore we are currently developing some implementations for various research groups in the fields of Metabolomics and Proteomics, with whom we are actively collaborating.

## Availability and requirements

• **Project name**: QTREDS (Quality and TRacEability Data System)

• **Project home page**: http://qtreds.crs4.it

• **Operating systems**: Platform independent

• **Programming languages**: Ruby, HTML, JavaScript

• **Server requirements**: Apache 2, Mongrel, Rails 2.3.2, MySQL 5.0

• **Web browser requirements**: Firefox 19+, Chrome, Safari 5+

• **License**: Free for academic use

## List of abbreviations

AJAX: Asynchronous JavaScript And XML; API: Application Programming Interface; BDD: Behavior-Driven Development; CRUD: Create Read Update Delete; CSGP: CRS4 Sequencing and Genotyping Platform; HTTP: HyperText Transfer Protocol. LIMS: Laboratory Information Management System; MVC: Model-View-Controller; ORM: Object-Relational Mapping; REST: Representational State Transfer; RDBMS: Relational Database Management System; SaaS: Software as a Service; SQL: Structured Query Language; XML: eXtensible Markup Language; XSD: XML Schema Definition; YAML: YAMAL Ain't Markup Language.

## Competing interests

The authors declare that they have no competing interests.

## Authors' contributions

PP wrote this paper, designed and implemented the system, GF designed and defined the architecture of the system coordinating development activities, LV contributed to the gathering of requirements from stakeholders and tested the application under real world conditions leading to major improvements in usability and responsiveness. PRT coordinated and supervised the entire project and contributed feedback and advice for this paper.

## Supplementary Material

Additional file 1 **Exome Library Preparation workflow**. The file illustrates an example of the structure of an experimental workflow that can be generated through the visual tool or directly in XML, provided it adheres to the syntactic rules defined in the XSD file. Click here for file

Additional file 2 **Workflow Grammar**. This file expresses the basic set of rules to which an experimental workflow written in XML must conform in order to be considered valid by QTREDS. Click here for file

## Declarations

The publication costs for this article were funded by Sardegna Ricerche.

## References

[B1] Nix DA Di Sera TL Dalley BK Milash BA Cundick RM Quinn KS Courdy SJ Next generation tools for genomic data generation, distribution, and visualization BMC Bioinformatics 2010 11 1 455 20828407 10.1186/1471-2105-11-455 PMC2944281

[B2] Stocker G Fischer M Rieder D Bindea G Kainz S Oberstolz M McNally J Trajanoski Z iLAP a workflow-driven software for experimental protocol development, data acquisition and analysis BMC Bioinformatics 2009 10 390 19941647 10.1186/1471-2105-10-390 PMC2789074

[B3] Morris JA Gayther SA Jacobs IJ Jones C A Perl toolkit for LIMS development Source Code Biol Med 2008 3 1 4 18353174 10.1186/1751-0473-3-4 PMC2322998

[B4] Prosad PJ Bodhe GL Trends in laboratory information system Chemom Intell Lab Syst 2012 118 187 192

[B5] Gibbon GA A brief history of LIMS Laboratory Automation & Information Management 1996 32 1 1 5

[B6] Bentley D Analysis of a Laboratory Information Management System (LIMS) 1999 http://www.umsl.edu/~sauterv/analysis/LIMS_example.html last visited: 10 May 2013

[B7] Abbott Company STARLIMS web-based platform for unified laboratory informatics 2013 http://www.starlims.com/en-us/solutions/lims/last visited: 10 May 2013

[B8] Sapio Sciences Examplar LIMS 2010 http://www.sapiosciences.com/LIMS/index.html last visited: 7 May 2013

[B9] LABVANTAGE Solutions Inc How differences in technology affect LIMS functionality, cost & ROI Tech rep 2011

[B10] Wood S Comprehensive Laboratory Informatics: A Multilayer Approach Am Lab 2007 39 16 20 23

[B11] Kent JT The Right LIMS Delivery Method 2009 http://www.bio-itworld.com/uploadedFiles/Bio-IT_World/Bio-IT_Issues/2009/Jan-Feb/LabAuto_supplement.pdf last visited: 10 May 2013

[B12] Bauch A Adamczyk I Buczek P Elmer F-J Enimanev K Glyzewski P Kohler M Pylak T Quandt A Ramakrishnan C Beisel C Malmstrom L Aebersold R Rinn B openBIS: a flexible framework for managing and analyzing complex data in biology research BMC Bioinformatics 2011 12 1 468 22151573 10.1186/1471-2105-12-468 PMC3275639

[B13] Truong CVC Groeneveld LF Morgenstern B Groeneveld E MolabIS - An integrated information system for storing and managing molecular genetics data BMC Bioinformatics 2011 12 1 425 22040322 10.1186/1471-2105-12-425 PMC3268772

[B14] Triplet T Butler G The EnzymeTracker: an open-source laboratory information management system for sample tracking BMC Bioinformatics 2012 13 1 15 22280360 10.1186/1471-2105-13-15 PMC3353834

[B15] Konertz R Open-LIMS 2010 http://www.open-lims.org last visited: 10 May 2013

[B16] Bika Lab Systems Bika Lims 2011 http://www.bikalabs.com/softwarecenter/bika last visited: 9 May 2013

[B17] Nelson EK Piehler B Eckels J Rauch A Bellew M Hussey P Ramsay S Nathe C Lum K Krouse K Stearns D Connolly B Skillman T Igra M LabKey Server: An open source platform for scientific data integration, analysis and collaboration BMC Bioinformatics 2011 12 1 71 21385461 10.1186/1471-2105-12-71 PMC3062597

[B18] Van Rossum T Tripp B Daley D SLIMS--a user-friendly sample operations and inventory management system for genotyping labs Bioinformatics 2010 26 14 1808 1810 20513665 10.1093/bioinformatics/btq271 PMC2894515

[B19] Goomedic 15 Free and Open source LIMS: Laboratory information management system programs and projects 2010 http://www.goomedic.com/15-free-and-open-source-lims-laboratory-information-management-system-programs-and-projects.html last visited: 10 May 2013

[B20] Sidore C Sanna S Kwong A Kang HM Cusano R Pitzalis M Zoledziewska M Maschio A Busonero F Lobina M Angius A Lyons R Terrier B Brennan C Atzeni R Mulas A Dei M Piras M Lai S Reinier F Berutti R Jones C Marcelli M Urru M Oppo M Schlessinger D Abecasis G F C Whole Genome Sequencing of 2100 Individuals in the founder Sardinian Population [abstract] Abstract volume of the 62nd Annual Meeting of The American Society of Human Genetics: 6-10 November 2012; San Francisco, USA 2012 76

[B21] Heinemeier Hansson D Ruby on Rails Framework 2004 http://rubyonrails.org/last visited: 8 May 2013

[B22] Oracle Inc MySQL: The world's most popular open source database http://www.mysql.com/last visited: 10 May 2013

[B23] Stephenson S Prototype JavaScript Framework 2005 http://prototypejs.org/last visited: 9 May 2013

[B24] Garrett J Ajax: A New Approach to Web Applications 2005 http://www.adaptivepath.com/ideas/ajax-new-approach-web-applications/last visited: 10 May 2013

[B25] Fuchs T script.aculo.us 2005 http://madrobby.github.io/scriptaculous/last visited: 9 May 2013

[B26] Taylor CF Field D Sansone S-A Aerts J Apweiler R Ashburner M Ball CA Binz P-A Bogue M Booth T Brazma A Brinkman RR Michael Clark A Deutsch EW Fiehn O Fostel J Ghazal P Gibson F Gray T Grimes G Hancock JM Hardy NW Hermjakob H Julian RK Kane M Kettner C Kinsinger C Kolker E Kuiper M Novère NL Promoting coherent minimum reporting guidelines for biological and biomedical investigations: the MIBBI project Nat Biotechnol 2008 26 8 889 896 18688244 10.1038/nbt.1411 PMC2771753

[B27] Rocca-Serra P Brandizi M Maguire E Sklyar N Taylor C Begley K Field D Harris S Hide W Hofmann O Neumann S Sterk P Tong W Sansone SA ISA software suite: supporting standards-compliant experimental annotation and enabling curation at the community level Bioinformatics 2010 26 18 2354 2356 20679334 10.1093/bioinformatics/btq415 PMC2935443

[B28] Palla P Frau G Vargiu L Rodriguez-Tome P QTreds: a flexible LIMS for omics laboratories [abstract] Embnet.journal 2012 18 Suppl B 38 39

[B29] Fielding RT Taylor RN Principled design of the modern Web architecture Acm T Internet Tech 2002 2 2 115 150

