# Supplementary material for: QTREDS: a Ruby on Rails-based platform for omics laboratories
Source: BMC Bioinformatics. 2014 Jan 10;15(Suppl 1):S13. doi: 10.1186/1471-2105-15-S1-S13 (PMC4015218; doi:10.1186/1471-2105-15-S1-S13)
Supplement: Additional file 2 — Workflow Grammar. This file expresses the basic set of rules to which an experimental workflow written in XML must conform in order to be considered valid by QTREDS. [file 1471-2105-15-S1-S13-S2.pdf]

```xml
<?xml version="1.0" encoding="UTF-8"?>
<xs:schema elementFormDefault="qualified"
        targetNamespace="http://www.w3schools.com"
        xmlns="http://www.w3schools.com"
        xmlns:xsi="http://www.w3.org/2001/XMLSchema-instance"
        xmlns:xs="http://www.w3.org/2001/XMLSchema"
        xmlns:ns="http://www.w3schools.com"
        xmlns:hfp="http://www.w3.org/2001/XMLSchema-hasFacetAndProperty">
  <xs:element name="protocol">
    <xs:annotation>
      <xs:documentation>Workflow Grammar</xs:documentation>
    </xs:annotation>

    <xs:complexType mixed="true">
      <xs:sequence>
        <xs:element ref="activities"></xs:element>
      </xs:sequence>

      <xs:attribute name="name" use="required"></xs:attribute>

      <xs:attribute name="version" use="optional"></xs:attribute>

      <xs:attribute name="input"></xs:attribute>

      <xs:attribute name="output"></xs:attribute>
    </xs:complexType>
  </xs:element>

  <xs:element name="activities">
    <xs:complexType mixed="true">
      <xs:sequence>
        <xs:element maxOccurs="unbounded" ref="activity"></xs:element>
      </xs:sequence>

      <xs:attribute name="number" use="optional"></xs:attribute>
    </xs:complexType>
  </xs:element>

  <xs:element name="activity">
    <xs:complexType mixed="true">
      <xs:choice maxOccurs="unbounded" minOccurs="0">
        <xs:element maxOccurs="1" minOccurs="0" ref="instrument"></xs:element>

        <xs:element maxOccurs="1" minOccurs="0" ref="input"></xs:element>

        <xs:element minOccurs="0" ref="dose"></xs:element>

        <xs:element minOccurs="0" ref="mix"></xs:element>

        <xs:element ref="label"></xs:element>

        <xs:element maxOccurs="1" minOccurs="0" ref="comment"></xs:element>
```

```
    </xs:choice>

    <xs:attribute name="description" use="optional"></xs:attribute>

    <xs:attribute name="id" use="required"></xs:attribute>

    <xs:attribute name="name" type="xs:string" use="required"></xs:attribute>
  </xs:complexType>
</xs:element>

<xs:element name="instrument">
  <xs:complexType mixed="true">
    <xs:choice maxOccurs="unbounded" minOccurs="0">
      <xs:element ref="input"></xs:element>

      <xs:element ref="result"></xs:element>

      <xs:element ref="dose"></xs:element>

      <xs:element ref="attribute"></xs:element>
    </xs:choice>

    <xs:attribute name="name" type="xs:string" use="required"></xs:attribute>

    <xs:attribute name="required" use="optional"></xs:attribute>

    <xs:attribute name="nickname" type="xs:string" use="optional"></xs:attribute>

    <xs:attribute name="category" type="xs:string"></xs:attribute>

    <xs:attribute name="id" type="xs:string"></xs:attribute>

    <xs:attribute name="default" type="xs:string"></xs:attribute>
  </xs:complexType>
</xs:element>

<xs:element name="mix">
  <xs:complexType>
    <xs:choice maxOccurs="unbounded" minOccurs="0">
      <xs:element ref="input"></xs:element>

      <xs:element ref="dose"></xs:element>

      <xs:element ref="instrument"></xs:element>
    </xs:choice>

    <xs:attribute name="name" type="xs:string" use="required"></xs:attribute>
  </xs:complexType>
</xs:element>

<xs:element name="result">
  <xs:complexType>
```

```
    <xs:sequence>
      <xs:element maxOccurs="unbounded" ref="attribute"></xs:element>
    </xs:sequence>

    <xs:attribute name="name" type="xs:string" use="required"></xs:attribute>

    <xs:attribute name="required" type="xs:boolean" use="optional"></xs:attribute>

    <xs:attribute name="multeplicity" type="xs:string" use="optional"></xs:attribute>
  </xs:complexType>
</xs:element>

<xs:element name="input">
  <xs:complexType>
    <xs:sequence>
      <xs:element maxOccurs="unbounded" ref="attribute"></xs:element>
    </xs:sequence>

    <xs:attribute name="name" type="xs:string" use="required"></xs:attribute>

    <xs:attribute name="required" type="xs:boolean" use="optional"></xs:attribute>

    <xs:attribute name="description" type="xs:string" use="optional"></xs:attribute>

    <xs:attribute name="id" type="xs:string"></xs:attribute>

    <xs:attribute name="multiplicity" type="xs:boolean" use="optional"></xs:attribute>
  </xs:complexType>
</xs:element>

<xs:element name="attribute">
  <xs:complexType mixed="true">
    <xs:attribute name="key" type="xs:string" use="required"></xs:attribute>

    <xs:attribute name="type" type="xs:string" use="required"></xs:attribute>

    <xs:attribute name="unit" type="xs:string" use="optional"></xs:attribute>

    <xs:attribute name="description" type="xs:string" use="optional"></xs:attribute>

    <xs:attribute name="extension" type="xs:string" use="optional"></xs:attribute>

    <xs:attribute name="required" type="xs:boolean" use="optional"></xs:attribute>

    <xs:attribute name="values" use="optional"></xs:attribute>

    <xs:attribute name="target" type="xs:string" use="optional"></xs:attribute>

    <xs:attribute name="function_name" use="optional"></xs:attribute>

    <xs:attribute name="function" use="optional"></xs:attribute>
```

```
      <xs:attribute name="hidden" use="optional"></xs:attribute>
    </xs:complexType>
  </xs:element>

  <xs:element name="dose">
    <xs:complexType mixed="true">
      <xs:all>
        <xs:element maxOccurs="1" minOccurs="0" ref="attribute"></xs:element>
      </xs:all>

      <xs:attribute name="name" type="xs:string" use="required"></xs:attribute>

      <xs:attribute name="source" type="xs:string" use="optional"></xs:attribute>

      <xs:attribute name="description" type="xs:string" use="optional"></xs:attribute>

      <xs:attribute name="category" type="xs:string" use="required"></xs:attribute>

      <xs:attribute name="multiplicity"></xs:attribute>

      <xs:attribute name="id"></xs:attribute>
    </xs:complexType>
  </xs:element>

  <xs:element name="user">
    <xs:complexType>
      <xs:attribute name="required" type="xs:boolean" use="required"></xs:attribute>
    </xs:complexType>
  </xs:element>

  <xs:element name="sample">
    <xs:complexType>
      <xs:attribute name="source" use="required"></xs:attribute>
    </xs:complexType>
  </xs:element>

  <xs:element name="comment">
    <xs:complexType>
      <xs:attribute name="use"></xs:attribute>

      <xs:attribute name="title" type="xs:string"></xs:attribute>
    </xs:complexType>
  </xs:element>

  <xs:element name="label"></xs:element>
</xs:schema>
```
